# Using a digital patient powered research network to identify outcomes of importance to patients with multiple myeloma

**DOI:** 10.1186/s41687-020-00242-5

**Published:** 2020-09-01

**Authors:** Katharine S. Gries, John Fastenau

**Affiliations:** Janssen Global Services, 700 US-202, Raritan, NJ 08869 USA

**Keywords:** Patient outcomes, Oncology, Social media

## Abstract

**Background:**

Social media platforms give patients a voice by allowing them to discuss their health and connect with others. These unfiltered and genuine reports offer direct access to what matters most to patients. Exploring the patient-reported outcomes discussed in these platforms reveal clinical insights and behavioral patterns of the real-world patient journey. This research study reviewed health-related quality of life (HRQoL) concepts reported by patients with multiple myeloma (MM).

**Methods:**

Data were obtained using the Belong.life patient-powered research network (PPRN) using social media listening methods. The analysis cohort consisted of adults diagnosed with MM who signed into the Belong.life platform by June 2018. Natural language processing and medical neural networks were utilized to extract text data to mine and scan for concepts using programmed algorithms. The textual review of the data was conducted on two levels: the over-arching concept of interest (broad symptom and impact classification) and the more specific symptom and impacts report. Concepts were analyzed descriptively and summarized by age, gender, context of report, and stage of disease/treatment journey.

**Results:**

Two hundred thirty patients with MM from the United States (52%), Israel (42%), Canada (3%), and 3% from Egypt, France, Greece, India, United Kingdom, and Australia were identified. A total of 57% were female and at account registration the median age was 57 years. A total of 126 patients had evaluable text data to search concepts being discussed. The PPRN platform identified 93% of the concepts from the conceptual model developed based on prior literature review. The most commonly reported symptoms were neuropathy, tiredness, nausea, back pain, fatigue, and bone pain. Back pain appeared as the most prominent symptom early in the disease and sometimes occurred prior to MM diagnosis. Tiredness, nausea, fatigue, and bone pain were frequently reported after MM diagnosis, with the start of treatment.

**Conclusion:**

Patient-oriented social media platforms, such as Belong.life, can capture and contribute to a holistic vision of concepts surrounding patients’ HRQoL. The ability to understand when a certain debilitating symptom appeared and to which sub-population of patients may allow for a personalized approach to treatment, improving adherence and quality of care as well as increasing patient well-being.

## Introduction

When diagnosed with a disease or initiated on a new treatment people will frequently turn to the internet and social media platforms for information and answers to their personal health questions. People may share stories about their medical treatments, their experiences with a medical condition, and their associated health-related quality of life (HRQoL) through social media posts and conversations with others. The data generated in forums, blogs and other online platforms are often anonymous but genuine, unfiltered, and instinctive offering direct access to patients’ experiences and insights that were previously unavailable for research purposes. This real-world data can be used to leverage patient-generated health data and because patients’ use these platforms to seek advice and share experiences it may enable greater capture of untoward clinical events [1]. Therefore, social media listening is a valuable tool for discovering patients’ unmet needs, understanding their experiences during the treatment journey, their symptoms, the impacts from the disease and/or treatment, and ultimately what matters to them the most.

Researching, sharing and discussing health information has become a prominent aspect in people’s lives. A national survey in the United States found that 72% adult internet users say they have searched online for information about a range of health issues; specific diseases and treatments [2]. Distinctively, 26% of internet users with a chronic health condition have read or watched someone else’s commentary or experience about health or medical issues online, 24% say they turned to others who have the same health condition while dealing with their illness, and 16% have gone online to find others who might share the same health condition [3]. Patients and caregivers can create a complete social-environment around their health condition and take an active role in sharing their health concerns and experiences. They can track health indicators or symptoms online, gather healthcare information, read about others’ personal health experiences and post comments, questions or share their insights from their personal disease journey and treatment path. Interactions on social media are based on trust and transparency and despite privacy concerns, many will share their medical information if the social environment they joined holds the potential to improve their health [4]. Literature reviews on social media taxonomy and use in cancer care; specifically among breast cancer survivors demonstrats how social media data was being used in health outcomes research and health communication [5, 6]. As part of this review, researchers found that while qualitative approaches can be used to explore these rich data databases, machine learning techniques have become more widely adopted as a method of analysis and review [5].

In realizing the importance of direct interaction among patients for purposes of supporting active health management, novel digital platforms such as Belong.life were developed. These digital platforms allow people to engage with other patients that share similar health conditions and discuss treatment options, disease symptoms, treatment side effects, and overall experiences. This engagement provides emotional support, social support and people feel heard by others living with a similar diagnosis. In the Belong.life platform, 84% of users agreed that they felt less alone when using the application [7]. These patient-powered research networks (PPRN) are driven by communities of engaged patients that are motivated to manage their illness, support and help others with similar health conditions and advance clinical research through sharing their data and experiences with researchers [8].

Social media data are rich sources of patients’ real-world health experience. Traditional data collection on the direct report of the patients’ health experience often involves a qualitative interview. Advantages of using social media platforms over qualitative interviews in understanding the patient journey and identify concepts of relevance to their experience are reduction in time/resources for patient recruitment, scheduling, and data collection, and the potential ability to elicit more concepts given the unbiased report and anonymity provided [9]. One disadvantage is the inability to probe on specific concepts or debrief on a topic of interest, unless the researcher becomes part of the social media conversation and engages with the patient. Also, when using social media data sources, the clinical diagnosis and other descriptive characteristics may be unavailable [9].

Multiple myeloma (MM) is a rare cancer of white blood cells called plasma cells with no cure, but survival has been significantly extended in the past two decades due to advances in treatment. Patients with MM are now living 10–15 years longer with the disease [10]. The complex organ damage in MM together with the side effects of treatment significantly impairs physical, psychological (cognitive and emotional), and social aspects of HRQoL. HRQoL refers to the subjective evaluation of one’s ability to perform usual tasks and their impact on one’s everyday physical, emotional, and social well-being [11]. Therefore, the purpose of MM treatments is to prolong survival while maintaining patients’ HRQoL.

In the current study, we utilized text data from social media conversations to identify concepts important to patients with MM. To characterize the usefulness of the PPRN for insight generation, concepts from existing MM patient reported outcome (PRO) instruments were compared to the disease-related symptoms and impacts identified through text review of the PPRN social media data.

## Methods

### Belong.life Patient-Powered Research Network (PPRN)

Belong.life is a patient education, engagement and disease journey navigation digital social media platform in oncology that was launched in 2015. It provides patients with a mobile phone application that incorporates personalized solutions such as: access to leading healthcare professionals who answer questions, a vast social network of patients with a similar cancer diagnosis and personalized information and notifications.

Access to the application is free for use and the engagement and data sharing in Belong.life is voluntary, anonymous and is contributed by patients and their caregivers. According to their preferences and needs, users can upload medical documents to their digital binder and share disease-related data with physicians and others in the professional and community groups. Therefore, the data accumulated in Belong.life may include healthcare provider summaries, test results, images, chats, and clinical trial matching requests making Belong.life unique compared to other PPRNs. Most of the data on the platform is social engagement data from the community and professional groups, inclusive of chat text and posts that are initiated by the users. The Belong.life is a Health Insurance Portability and Accountability Act (HIPAA) compliant application and all information is saved securely to protect patients’ privacy.

### Patient privacy and ethical review board

Belong.life ensures the personal health information of its users is de-identified to protect their privacy. Upon registration to the application, users choose an anonymous username and approve the terms of use, the privacy policy and the usage of their de-identified, aggregated data for research purposes. The research protocol was reviewed by Quorum Review Independent Review Board (Seattle, WA) and received an exemption determination (Quorum Review File #33089; February 2018).

### Study sample

The study sample was defined using the following eligibility criteria: Diagnosed with MM, age ≥ 18 years at time of account registration, from any country, any gender, signed into the Belong.life platform by June 2018, and having evaluable patient-provided text data. MM patients’ diagnoses were corroborated through use of an algorithm and manually verified by researchers to confirm the diagnosis using available data within the PPRN. To be included in the analysis set the patient had to contribute text data. The algorithm searched the database for the following criteria: mentioned MM diagnosis in post, engaged with Belong.life support team and validated MM diagnosis, uploaded medical records that indicated MM diagnosis/treatments, or shared treatment sequence characteristics of MM.

### Data acquisition

This study was a retrospective analysis on data collected in the Belong.life PPRN; data collected through social listening, the process of monitoring the text conversations without intervening or becoming part of the conversation. The platform generates data across 4 dimensions: time, medical, emotional, and behavioral data. Using natural language processing (NLP) and medical neural networks, large extracts of structured and unstructured data into a structured 4-dimensional data lake were performed [12]. The patient-provided text data, used in this study, was collected from the time patients logged into the Belong.life application and was composed of all the data generated organically from patients’ usage of the application without any active interventions collecting specific data such as questionnaires or medical information/documents provided by clinicians.

**Fig. 1:**
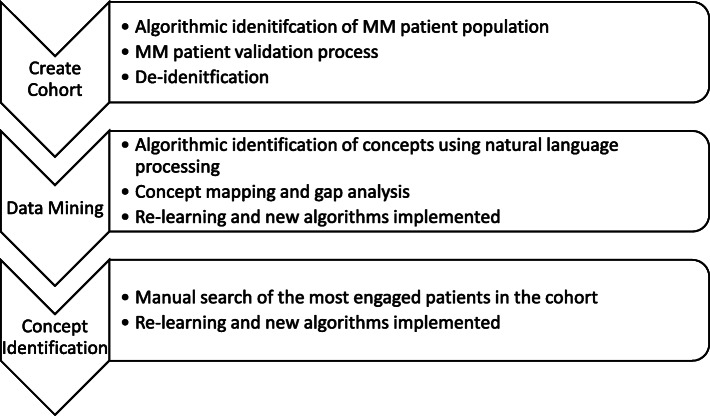
Data Acquisition process from the Belong.life Patient Powered Research Network

**Fig. 2:**
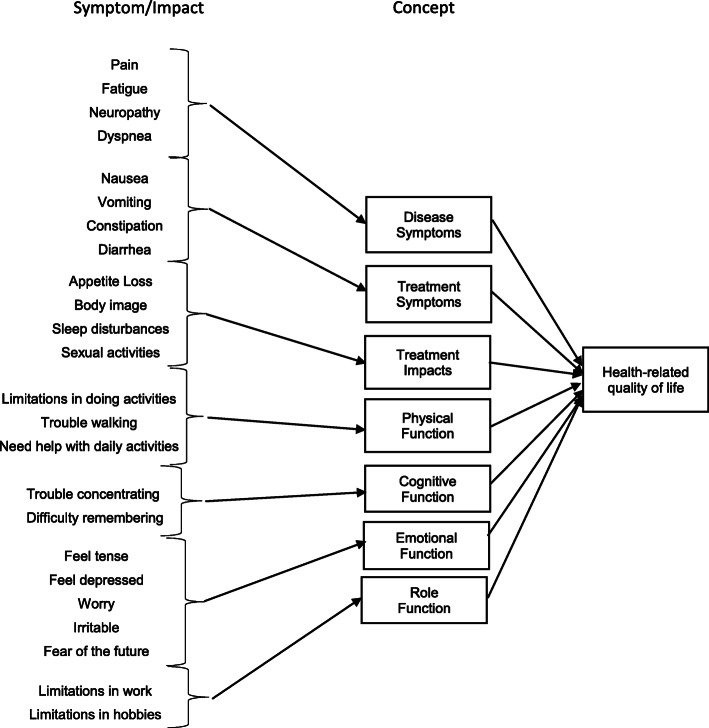
Conceptual Model. Conceptual model developed based on symptoms and impacts included in existing PRO instruments, validated for use as an endpoint in MM clinical trials. Not an exhaustive report of concepts, but a representation to initiate the data search and creation of search algorithm

Creation of the data analytical file is described in Fig. 1. The first step was to define and create the cohort using an algorithm to identify users with MM and upload their data into a dashboard for analysis. NLP was used to mine the textual data in the analysis set and create concept maps. The data mining algorithms were created to search for MM concepts. These concepts included disease-related and treatment-related symptoms and impacts identified through the development of a conceptual model created a priori to the data analysis. Concepts were pulled from the European Organization of Research and Treatment of Cancer Quality of Life Questionnaire core-30 item (EORTC QLQ-C30) and the EORTC QLQ Multiple Myeloma Module (EORTC QLQ-MY20), existing PRO instruments validated in patients with MM: Disease Symptoms, Treatment Symptoms, Treatment Impacts, Physical Function, Cognitive Function, Emotional Function, and Role Function, and HRQoL (Fig. 2). After a review of the data output, new algorithms were created for system re-learning to capture symptoms and impacts not previously identified from the conceptual model in Cycle 1.

The textual review of the data was conducted on two levels: the over-arching concept of interest (broad symptom and impact classification) and the more specific symptom and impact terms. For example, “disease symptoms” was searched as the concept and then “pain” was searched as a symptom in that disease symptom classification. We explored the symptoms and impacts endorsed by MM patients and characterized their frequency. We further evaluated the frequency of the leading symptoms and impact reported by MM patients overall and at different junctions in their treatment journey, reported by the patient, reflecting their clinical, physical, cognitive, emotional and social status at those timepoints.

### Data analysis

Descriptive summary statistics were performed on available demographic and clinical data for the study sample. An analysis of the key symptoms and impacts was performed on the analysis set to summarize the data by age, gender, and the context of the reported concept appearance overall as well as at the stage of the disease/treatment journey.

## Results

### Demographics

The study sample consisted of 230 patients with MM; patients were from the United States (52%), Israel (42%), Canada (3%) and other countries (3%; from Egypt, France, Greece, India, United Kingdom and Australia). From the study sample, 126 patients had evaluable text data and were included in the analysis set. These were the patients who were engaged and active in the social media conversations by writing posts or participating in chats. There were slightly more women (57%) than men (43%). At account registration, the median age of the study sample was 57 years with 75% of the sample being older than 50 years, and 43% older than 60 years of age.

### Concepts identified

**Fig. 3:**
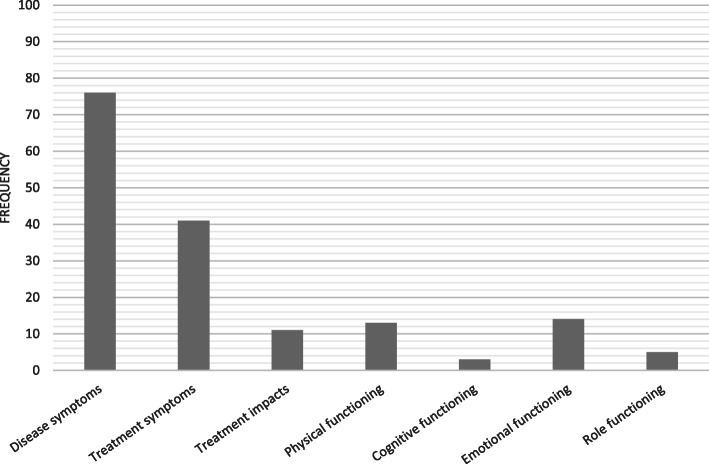
Frequency of Concepts Discussed by Patients

**Fig. 4:**
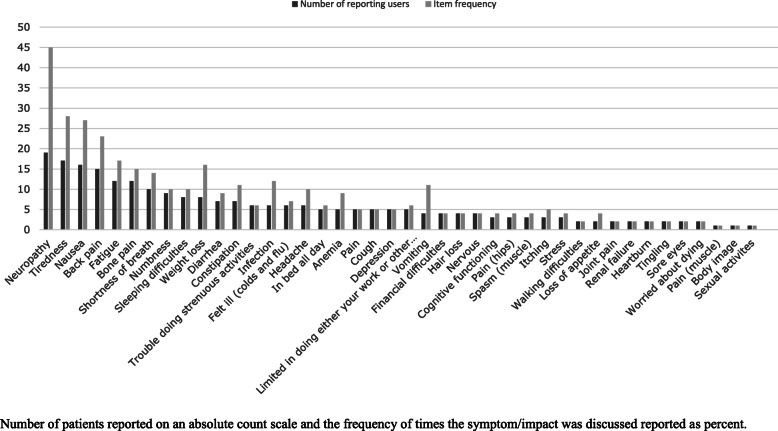
Number of Patients and Frequency of Symptoms and Impacts Reported by Patients. Number of patients reported on an absolute count scale and the frequency of times the symptom/impact was discussed reported as percent

The PPRN platform identified 93% of the concepts in the conceptual model; all of the symptom and impact concepts in the conceptual model were identified but not the general HRQoL term. The most frequently reported concepts included disease symptoms and symptomatic treatment effects (Fig. 3). A detailed analysis of the symptoms was performed on the following: neuropathy, tiredness, nausea, back pain, fatigue and bone pain as these were the most commonly reported by patients with MM (Fig. 4 and Table 1). Patients often mentioned symptoms and impacts multiple times in their text dialogue resulting in item frequencies higher than the number of reporting patients (Fig. 4).

**Table 1:** Most Commonly Reported Symptoms

	Age range	Gender		Context/Reason of Discussion			Example Patients’ Quotes
		Female	Male	Disease symptom	Side effect of treatment	Unknown reason	
Neuropathy (*n* = 19)	53–64	67%	33%	0%	74%	26%	“Neuropathy was soooo bad some days I just could not get out of bed” “I have severe neuropathy from treatment”
Tiredness (*n* = 17)	47–71	63%	37%	6%	65%	29%	“I am 2 weeks out of stem cell transplant. I still feel weak and tired all the time” “I don’t tolerate it well. Makes me feel very tired”
Nausea (*n* = 16)	33–69	69%	31%	6%	56%	38%	“Besides tiredness and nausea, I’m fine” “I only has a little bit of vomiting and nausea after the procedure”
Back pain (*n* = 15)	53–61	88%	12%	40%	53%	7%	“Back pain and feeling lethargic are the only symptoms so far” “She had pain in back for 2 years before I insisted on X-ray to find a tumor had encompassed her T5 vertebra and crushed it”
Fatigue (*n* = 12)	38–71	43%	57%	8%	75%	17%	“The fatigue for him lasted longer but he was back to work in 3.5 months for half days” “My side effects are almost continuous dizziness, shakiness, extreme dry eyes, fatigue”
Bone pain (*n* = 12)	33–75	67%	33%	33%	25%	42%	“I am having bad bone pain all over” “Not so great because of bone pain”

Thorough characterization of the most commonly reported symptoms by age range, gender distribution, stage of disease, and the context of report (attributed by the patients as disease symptom or symptomatic treatment effect) is provided in Table 1. Neuropathy was the most discussed symptom with 15% of patients reporting (*n* = 19). Patients reporting neuropathy had an age range of 53–64 years with a majority being women (67%). The most prominent context in which this symptom appeared was discussed as a side effect of treatment (74%). Patients specifically mentioned neuropathy in hands and feet because of treatment and mentioned that it can be severe and confine them to bed. Most patients reporting tiredness (13%; *n* = 17) were women (63%) with a wider range in age (47–71 years) than those reporting neuropathies. Tiredness was associated with pharmacologic treatment and recovery from treatments such as stem cell transplant. Patients reported feeling tired for prolonged periods of time. Nausea was reported by patients in the age range of 33–69 years (13%; *n* = 16) and was described as a side effect of treatment in 56% of the patients. This specific item had limited physical disabling impact as reported in the texts of patients. In contrast, back pain (12%; *n* = 15) had different reported characteristics than the other symptoms. The patient group reporting back pain had the narrowest age range (53–61 years) and was predominately reported by women (88%). 40% of back pain was the highest frequency among disease-related symptom reports. Fatigue and bone pain were each reported by 10% of patients (*n* = 12) and while fatigue was predominantly mentioned as a side effect of treatment (75% of the reports), only a quarter of the bone pain reports were mentioned as such. Bone pain, like back pain, was often reported as being associated with the disease (33%).

The leading symptoms were summarized according to when they were reported in the patient’s disease journey (Table 2). Back pain appeared as the most prominent symptom early in the disease course and sometimes occurred prior to diagnosis, possibly being the reason for patients to seek medical care. Tiredness, fatigue, nausea, back pain, and bone pain were discussed during the period of diagnosis. Neuropathy was frequently discussed by patients after experiencing a relapse of their MM.

**Table 2:** Symptom Discussed by Stage of Disease Journey

	Before Diagnosis	Newly Diagnosed; Before Treatment	After Stem Cell Transplant	Remission	Relapsed	Not provided
Neuropathy (*n* = 19)	0	6 (32%)	2 (10%)	2 (10%)	7 (37%)	2 (10%)
Tiredness (*n* = 17)	0	9 (53%)	2 (12%)	3 (18%)	1 (6%)	2 (12%)
Nausea (*n* = 16)	1 (6%)	8 (50%)	3 (19%)	0	0	4 (25%)
Back Pain (*n* = 15)	4 (27%)	7 (47%)	1 (7%)	0	1 (7%)	2 (13%)
Fatigue (*n* = 12)	1 (8%)	7 (58%)	0	0	1 (8%)	3 (25%)
Bone Pain (*n* = 12)	1 (8%)	4 (33%)	2 (17%)	0	1 (8%)	4 (33%)

### Identification of new concepts

Two concepts were identified that were not part of the pre-defined conceptual model: use of alternative medicine (*n* = 25) and lifestyle changes (*n* = 20). The more common aspects of alternative medicine discussed were cannabis use, alkaline diet, and turmeric curcumin use. Lifestyle changes included engaging in sports, consuming organic foods and meditation.

## Discussion

This study successfully explored whether social listening through a unique patient-oriented social media platform could identify existing and new concepts that are of importance to MM patients and then to summarize the concepts by patients’ characteristics and when these concepts appeared during the patients’ disease journey. The popularity of social media platforms has highlighted the potential of online interactions within patient communities and between patients and care providers as rich data sources for research [13]. It is recognized that patient-generated data from PPRNs, although regarded as nontraditional sources of biomedical and healthcare data, hold tremendous potential in assembling a holistic picture of the patients’ journey [14]. This is even more so for Belong.life, that developed a patient-centered platform in oncology allowing direct patient-to-patient and patient-to-healthcare professional communication and facilitates a multi-dimensional data sharing that creates a holistic view of a patient’s journey.

Our study is the first instance of identification of concepts important to patients with MM using the Belong.life PPRN. Given the high concordance of concepts identified in the database with concepts already known through existing PRO instruments [15, 16], we believe the use of this platform in MM research supports insight generation on what matters most to patients. Using a PPRN such as Belong enabled research activities to be completed efficiently because of the readily available sample of patient level data. Analysis of PPRNs for insight generation can provide complementary foundational research for concept elicitation required in new PRO instrument development and PRO measurement strategies. Traditional qualitative interviews and focus groups conducted with semi-structured interview guides allow for in-depth exploration into the patients journey and health state and hypothesis supporting data generation. This differs and complements the social listening and text review performed in the PPRN that has the potential to expose a breadth of concepts and patient insights. Additionally, in the context of PRO instrument development a review of the PPRN social media posts can provide insights to be further probed in a qualitative interview.

Our research successfully captured all of the specific concepts known and included in the conceptual model after removing the generic HRQoL search term. While the new concepts identified, use of alternative medicine and lifestyle changes, do not reflect specific disease or treatment-related symptoms, these concepts do provide context into the patient’s social and emotional state and their everyday life and struggle with MM. Our study illustrates the successful process of extracting large amounts of data about a patient’s health state, from social media texts, using NLP on unstructured clinical documents and medical social media to conduct meaningful research. Generating actionable health insights through social media remains a challenge due to typographic errors, ungrammatical structures, colloquial language and lack of context in addition to the inherent complexity of medical text [17]. Despite these difficulties our algorithms were able to detect most of the MM concepts included in existing PRO instruments after one cycle of data review.

In general, the technological nature of patient’s social engagement platforms such as Belong.life are assumed to have a certain bias towards younger patients due to the nature of its technology. In the case of MM, literature indicates that the median age of MM patients is 66–70 years [18] while the median age of the MM cohort in the PPRN was 57 years, suggestive of a limitation in the generalizability of research findings. The retrospective data mining approach is tapping into unbiased data in a non-interventional matter and therefore reflects the issues that truly bother patients or experiences that affected them the most at any given junction in their journey. We were able to show that relevant and important patient-reported concepts for measure in PRO instruments are being discussed by patients on social media platforms and that textual data can be extracted for thematic analysis. A limitation of our analysis was approximately half of our sample had evaluable patient-provided text data and was included in the analysis set. Researchers need to be cognizant that patient’s level of engagement with PPRNs varies which may bias findings depending on the study’s objective. The Belong Medical Network classifies patients based on 3 profiles: proactive users, passive users, and knowledge driven users. Proactive users are the patients who contribute to the social media data source; whereas the passive users log in to read posts and the knowledge driven users are there to collect data. For the purposes of our study, the level of patient engagement likely had limited impact on the insights generated.

Further developments in NLP technologies will advance the performance of medical social network data mining. More research is needed to explore the use of PPRNs, like Belong.life, to address research hypothesis testing. And while a high concordance of concepts from existing PRO instruments were identified in this social media review, use of social media data mining does not replace traditional qualitative interviews as an integral part of PRO instrument development for use in medical research.

As the Belong.life platform is a constantly growing community with patients continuous reporting, it allows not only a growing evidence for detected concepts in many patients but also presents the possibility of following specific concepts through the full patient journey. Focusing on symptom report related to patient characteristic and treatment pathway has tremendous implications in understanding, more specifically, what are the parameters that are associated with specific HRQoL outcomes, what are the meaningful concepts to measure, and will allow a more personalized approach to treating these patients. Combined with the conventional concept elicitation methods [19, 20] this could lead to a more comprehensive identification, validation and evaluation of new concepts to support PRO research.

## Conclusions

Patient-oriented social media platforms, such as PPRN like Belong.life, can capture and contribute to a holistic vision of concepts influencing patients reported HRQoL. The unique approach of data mining of text content provided by patients can inform researchers on the important concepts to measure and when to capture them during a patient’s disease journey. Social listening can be utilized as a method in PRO research and for further understanding of how concepts may change throughout the stages of a patient’s journey.

## Data Availability

The data that support the findings of this study are available from Belong. Life but restrictions apply to the availability of these data, which were used under license for the current study, and so are not publicly available. Data are however available from the authors upon reasonable request and with permission of Belong.Life.

## Abbreviations

HIPAA: Health Insurance Portability and Accountability Act
HRQoL: health-related quality of life
MM: Multiple myeloma
NPL: Natural language processing
PPRN: Patient powered research network
PRO: Patient reported outcome
